# Limited Availability and Higher Cost of Gluten‐Free Foods Continue in the United Kingdom: A Comparative Follow‐Up Over More Than a Decade

**DOI:** 10.1111/jhn.70209

**Published:** 2026-02-02

**Authors:** Harrison McInnes, Lea Klapan, Umara Moore, Jaspreet Singh, Kevin Whelan

**Affiliations:** ^1^ Department of Nutritional Sciences King's College London London UK

**Keywords:** availability, coeliac disease, cost, food access, gluten, gluten‐free diet

## Abstract

**Background:**

Coeliac disease requires strict lifelong adherence to a gluten‐free diet, although adherence is challenged by the limited availability and higher cost of gluten‐free foods. The demand for gluten‐free foods has increased over recent years, yet its impact on availability and cost is unknown. This study aims to assess the availability and cost of gluten‐free food across diverse retail outlets and compare changes over 11 years.

**Methods:**

Replicating the same methodology as our previous study in 2010, the availability and cost of 20 foods (10 wheat‐based, 10 everyday foods) were assessed in 30 stores across a diverse range of London boroughs and compared over an 11‐year period. For each of the 20 foods, the cost of four products were selected (branded gluten‐free, cheapest gluten‐free, branded standard, cheapest standard) and compared.

**Results:**

Availability of the 20 foods in 2021 was generally limited, with an average of 7.6 (SD 5.7) gluten‐free foods per store (38.2% of foods surveyed). Regular supermarkets in particular saw a reduction in availability over time from 18.0 (SD 2.0) of the 20 foods (90% of foods) available in gluten‐free versions in 2010 decreasing to 14.0 (SD 1.4, 70%) in 2021 (*p* = 0.008). Gluten‐free foods were more expensive than gluten‐containing counterparts. In terms of secular trends, the 2021 cost generally exceeded inflation‐adjusted estimates; however, the ratio between the cost of gluten‐free and gluten‐containing foods declined over the 11 years.

**Conclusions:**

Over 11 years in the UK, gluten‐free food availability remained limited and more costly. Any impact of sustained limited availability and higher cost on adherence to a gluten‐free diet should be addressed and interventions to overcome these issues should be tested.

## Summary

- Gluten‐free foods are essential in the management of coeliac disease.
- On average, only 7.6 gluten‐free foods (out of 20 foods surveyed) were available in a range of stores in the UK, a situation that has not improved in over a decade.
- Regular supermarkets saw a reduction in the availability of gluten‐free foods during this time.
- Gluten‐free foods continue to be more expensive than their standard counterparts, often exceeding inflation, although the gap has declined over time.

## Graphical abstract

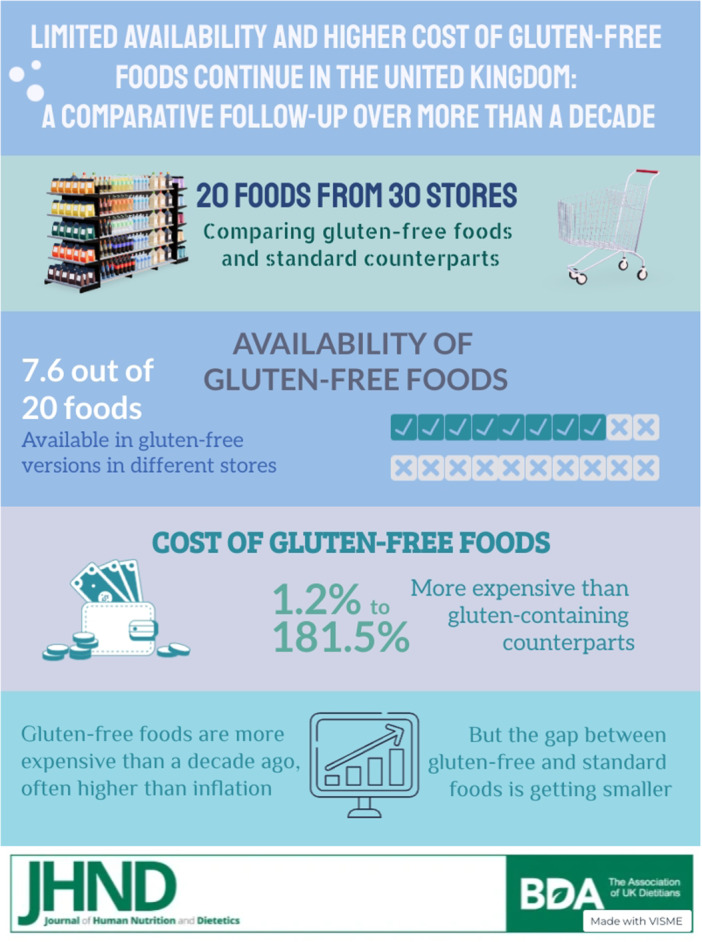

## 1 Introduction

Coeliac disease is a chronic autoimmune disorder triggered by exposure to dietary gluten in genetically predisposed individuals. Untreated, coeliac disease leads to small intestinal inflammation characterised by mucosal damage, villous atrophy, infiltration of lymphocytes into the epithelium, and increased density and depth of the crypts [1, 2]. Manifestations range from severe gastrointestinal symptoms, including malabsorption, diarrhoea, constipation, vomiting, anorexia, and weight loss to chronic complications including extraintestinal symptoms such as anaemia, osteoporosis, arthritis, as well as neurological, endocrinological, musculoskeletal, psychological, oral and dermatological disorders [1, 2].

Coeliac disease affects approximately 1% of the UK population, 1%–2% globally, and up to 10%–20% among first‐degree relatives [3, 4, 5]. The prevalence of coeliac disease has increased significantly, with a fourfold rise observed between 1990 and 2011, and a global incidence growth of approximately 7.5% over recent decades [5, 6].

Lifelong adherence to a gluten‐free diet remains the only treatment for coeliac disease. The gluten‐free diet is effective in managing symptoms and preventing complications however nutritional challenges occur in some, with evidence of reduced dietary fibre intake, high glycaemic index carbohydrate sources, and inadequate essential micronutrients, including vitamins D, E, and B‐complex, as well as minerals such as iron, iodine, zinc, selenium, manganese and calcium [7, 8, 9, 10, 11, 12, 13].

There are also psychosocial challenges with maintaining a gluten‐free diet. People with coeliac disease can experience anxiety and fear of eating, and recent evidence of impaired food‐related quality of life [7, 14]. These factors can adversely affect eating behaviours and therefore food and nutrient intake, with some evidence of an increased risk of disordered eating patterns [14, 15, 16].

A major challenge to sourcing, purchasing, adhering to, and enjoying a gluten‐free diet is the availability and cost of gluten‐free foods [13]. For example, one study reported that only 59.7% of 67 retail outlets in the United Kingdom (UK) stocked one of 10 gluten free alternatives, all of which were on average 4.1 times more expensive than their gluten‐containing counterparts [17]. Availability varied by type of store, being highest in regular and premium/quality supermarkets, particularly within larger stores, whereas health food retailers offered a limited selection, and budget or convenience/local stores stocked no gluten‐free items [17]. Another study in the UK reported that 91% of the gluten‐free products surveyed were higher in cost, with gluten‐free bread and bread rolls costing 400% and 379% more, respectively, than standard gluten‐containing equivalents [18]. Whilst availability was higher in certain store categories and online retail platforms, cost remained a significant barrier.

Preliminary findings suggest that limited availability and higher cost of gluten‐free foods may not be improving over time. One longitudinal study reported increased availability of gluten‐free foods in the UK over the period from 2015 to 2019; however, this was accompanied by a rise in price ratio of gluten‐free to gluten‐containing equivalents from 3.2 to 4.1 [19]. Another longitudinal study reported an increase in availability of gluten‐free products in the United States (US) over a decade, highlighting a 183% cost differential compared to gluten‐containing equivalents, a value that had declined from 240% in 2006 [20].

These findings regarding availability and cost align with the experiences of people with coeliac disease who report improvements in the availability of gluten‐free foods yet consider them to be prohibitively expensive and disproportionate [21]. A recent survey conducted in the UK recognised a poorer gluten‐free dietary adherence in those who perceived that the cost of gluten‐free food restricts their dietary intake, of which over half of the participants with coeliac disease agreed [22]. Similarly, poorer adherence was observed in those who had to undertake additional travelling to purchase gluten‐free food [22]. Disparities in availability and cost disproportionately impact individuals from lower socioeconomic backgrounds, as well as elderly and disabled people affected by coeliac disease [20, 21]. Data from the past decade indicated that maintaining strict adherence to a gluten‐free diet continues to pose significant challenges for people with coeliac disease, for reasons including the quality and organoleptic properties of gluten‐free foods as well as their limited availability and higher cost [9, 23].

In 2010, we examined the cost and availability of gluten‐free foods, including both wheat‐based and everyday gluten‐containing foods across a wide range of store types in the UK [24]. We surveyed 20 foods in 30 different stores, across five different store categories in Greater London [24]. In view of increasing numbers of people diagnosed with coeliac disease [5, 6], increasing use of gluten‐free foods by those without coeliac disease [19] and continued reports of gluten‐free food availability and cost being barriers to adherence to a gluten‐free diet [18, 19, 25], we wanted to examine whether secular changes in availability and cost of gluten‐free foods had occurred since our original study. Therefore, the aim of the current study is to investigate the availability and cost of gluten‐free foods across a wide range of retail outlets, and to compare changes over an 11‐year interval.

## 2 Materials and Methods

This study, undertaken in 2021, investigated the availability and cost of the same 20 foods at the same 30 stores (where possible) and using the same methodology as our previous study conducted over a decade earlier in 2010 [24]. Ethical approval was not required for this study as there were no human participants.

### 2.1 Food Items

The cost and availability of the same 20 food items were surveyed in each store. These foods continued to be regarded as everyday staple foods in the UK over this period. They consisted of 10 wheat‐based foods (where wheat is a major ingredient i.e. bread, bread rolls, breakfast cereal, pasta, flour, cream crackers, sweet biscuits, pastries, pizza base, whole cake) and 10 common everyday foods that frequently contain wheat or gluten as an added ingredient (stock cubes, gravy granules, barbecue sauce, brown sauce, soy sauce, frozen beef burgers, frozen sausages, frozen chicken ready meal, fish fingers, shepherd's pie).

### 2.2 Stores

The cost and availability of these 20 food items were surveyed across 30 different stores throughout London, United Kingdom. The stores were divided into five categories, defined as follows: (i) Quality supermarkets (wide variety and range of selected quality foods, many specialist products, generally higher cost); (ii) Regular supermarkets (wide variety and range of both quality and budget foods, competitive cost); (iii) Budget supermarkets (limited variety and range of low cost food, limited specialist products); (iv) Health food shops (wide range of foods for specialist dietary needs, including many organic products); and (v) local (corner) shops (general product lines, mostly essential and convenience foods, small stores, often independently run and locally situated). Although there is no consensus on which stores meet each definition, nine clinical and academic dietitians previously reached agreement on store classification to determine the allocation of six stores within each category [24]. The five store categories were chosen to represent a diverse range of shopping outlets, reflecting consumer food purchasing preferences across different population groups with varying economic, demographic and accessibility circumstances. Supermarkets and health food stores in particular have been included as these are stores where people with coeliac disease frequently purchase gluten‐free foods [26].

For each of the five categories, six stores (total of 30 stores) were selected that were distributed geographically across London to account for variations in cost and availability among local populations, ensuring that for each store category there was at least one in North, East, South, and West London and from a range of areas within these. Furthermore, stores within the supermarket categories (regular, quality, budget) were selected to be of similar size to control for variations in stock and product availability between stores of different sizes.

For the 2021 round of data collection, the aim was to visit the identical 30 stores originally surveyed in our previous study [24]. An online search was conducted prior to store visits, which identified that 24 of the original stores were still open. Three stores had permanently closed (two budget supermarkets and one local shop), two could not be located online (both local shops), and one was temporarily closed for refurbishment (quality supermarket). In these six cases, stores from the same category that were of similar size and located nearby were selected as equivalents for the survey.

Although online food purchasing has increased substantially in the intervening years, it was not included in our follow‐up study to ensure methodological alignment with the original study in 2010 to allow for direct comparisons. However, the researchers acknowledge that the growing prevalence of online retailing now represent a significant source of gluten‐free foods, which may influence the availability and cost in contemporary contexts [17, 18, 20, 27].

### 2.3 Survey Procedure

As in our procedure in 2010, each shop was visited in person over a 2‐month period to reduce differences due to seasonal stock variations. Following verbal permission from a store‐worker, a survey of the availability and cost of the 20 different foods was undertaken. In each case, to confirm whether a product was gluten‐free (or gluten‐containing), the Coeliac UK directory of gluten‐free foods was referenced, or alternatively, a nutritionist manually examined the product ingredient labels for gluten sources [28].

For availability, the survey consisted of recording the number of gluten‐free versions of each of the 20 foods. For cost, the cost and weight of both gluten‐free and standard versions of the 20 food items were recorded. This involved selecting four products, where available, (branded gluten‐free, cheapest gluten‐free, branded standard, cheapest standard), which resulted in recording the cost and weight of up to 80 products in each store. When selecting gluten‐free and standard versions, attempts were made to select from comparable products (e.g., comparable brand, comparable pack size) in order to ensure that cost comparisons were not disproportionately affected by the greater availability of ‘quality' or ‘basic’ products in the standard versions. Additionally, for the brands of gluten‐free foods recorded in 2010, identical brands were re‐recorded in 2021 (where available) to enable fair calculation of secular trends that were not impacted by inter‐brand cost variations.

The brand name, weight/package size and cost were recorded. The cost was expressed in British sterling, with the conversion rates at the time of 2021 data collection being £1 (100 pence) = US$1.42 and €1.16. To standardise comparisons between products with different weights and package sizes, the cost of foods was expressed as pence per 100 g where possible.

### 2.4 Statistical Analysis

Statistical analysis was conducted using SPSS, version 27.0 (SPSS Inc., Chicago, IL, USA). Data for availability and cost were visually inspected, and in general were normally distributed. Parametric analysis was therefore conducted throughout to enable the use of consistent data summaries (i.e., mean, SD) between different food items. A *p*‐value of ≤ 0.05 was considered statistically significant.

Availability was calculated as both whether a gluten‐free version was available (yes/no), as well as the number of gluten‐free versions available. Cost was calculated as the mean of the branded and cheapest versions. A one‐way analysis of variance (ANOVA) was conducted to assess differences in the availability and cost of gluten‐free foods across the five store categories, and where significant, this was followed by an independent samples *t*‐test and Bonferroni post hoc correction to compare between two store categories.

A paired *t*‐test was performed to compare the availability and cost of gluten‐free foods between 2010 and 2021, for identical products and stores (or their substitutes). Increase in cost over time is common due to financial inflation, therefore the Consumer Price Index (CPI) was used to estimate the anticipated cost of a product adjusted for the inflation anticipated price over time. To calculate the expected cost of foods in 2021, the food‐specific CPI weights for 2010 and 2021 were applied, which were 108 and 114 (parts per 1000), respectively [29]. Thus, the ratio of the 2021 and 2010 price indices (114/108) represents an average inflation rate of foods of 1.05% over this period. Therefore, as well as reporting costs in both 2010 and 2021, we were able to report the anticipated inflation‐adjusted cost of each product from 2010 should it have followed anticipated inflation for an average food product by 2021 [29]. To compare the price differential between gluten‐free and standard (gluten‐containing) products in 2010 and 2021, a ratio was calculated by dividing the average cost of a gluten‐free product (the average of the cheapest and branded versions) by the average cost of a standard gluten‐containing product (the average of the cheapest and branded versions) in both 2010 and 2021. A paired t‐test was performed to assess the significance of the differences between ratios over this period.

## 3 Results

### 3.1 Availability of Gluten‐Free Foods in 2021

Availability of the 20 foods across the five store categories was generally limited, with an average of 7.6 (SD 5.7) of the 20 (38.2%) gluten‐free foods available per store. There was considerable variation in availability across store categories. On average, regular supermarkets stocked 14.0 (SD 1.4) of the 20 foods (70% of foods) in gluten‐free versions, followed by 10.3 (SD 2.3, 51.7%) in health food shops, 7.8 (SD 5.1, 39.2%) in quality supermarkets, 5.2 (SD 5.8, 25.8%) in budget supermarkets and 0.8 (SD 0.8, 4.2%) in local shops (*p* < 0.001).

When considering the number of versions of individual foods, the availability of gluten‐free versions of wheat‐based products, which ranged from an average of 0.4 (fruit pies) to 11.4 (sweet biscuits) was generally greater than for everyday foods, which ranged from 0.0 (brown sauce, frozen chicken sauce meal and shepherd's pie) to 1.5 (stock cubes) (Table 1).

**Table 1:** Availability (number of different versions) of gluten‐free foods according to store category in 2021.

Availability, mean (SD)	Overall	(a) Quality supermarket	(b) Regular supermarket	(c) Budget supermarket	(d) Health food shops	(e) Local shops	*p*
Bread	5.0 (7.2)	4.3 (6.6)	13.8 (7.4)^c^^,^ e	2.8 (4.7)^b^	4.2 (7.2)	0.0 (0.0)^b^	**0.005**
Bread rolls	1.8 (2.7)	2.0 (2.5)	5.2 (2.4)^d^^,^ e	1.5 (3.2)	0.3 (0.5)^b^	0.0 (0.0)^b^	**0.002**
Breakfast cereals	2.8 (3.3)	1.2 (1.3)^d^	5.3 (2.9)^d^	1.5 (2.3)^d^	6.0 (3.6)^a^^,^ c ^,^ e	0.0 (0.0)^b^^,^ d	**< 0.001**
Pasta	5.8 (8.9)	2.7 (2.7)^d^	6.3 (2.3)^d^	1.3 (2.1)^d^^,^ e	18.5 (13.3)^a^^,^ b ^,^ c ^,^ e	0.0 (0.0)^d^	**< 0.001**
Flour	3.6 (5.3)	1.8 (2.3)^d^	2.7 (1.9)^d^	1.0 (2.0)^d^	11.8 (6.7)^a^^,^ b ^,^ c ^,^ e	0.7 (0.8)^d^	**< 0.001**
Cream crackers	4.8 (5.2)	4.2 (4.5)	6.7 (0.8)	3.5 (4.5)	9.5 (7.3)^e^	0.0 (0.0)^d^	**0.012**
Sweet biscuits	11.4 (12.5)	10.0 (8.6)	21.8 (3.9)^e^	11.8 (18.2)	13.3 (14.0)	0.0 (0.0)^b^	**0.039**
Fruit pies	0.4 (1.8)	0.0 (0.0)	2.2 (3.9)	0.0 (0.0)	0.0 (0.0)	0.0 (0.0)	0.145
Pizza bases	0.5 (0.6)	0.3 (0.5)	1.0 (0.0)	0.5 (0.5)	0.8 (0.8)	0.0 (0.0)	**0.010**
Whole cake	1.6 (3.1)	1.5 (2.8)^d^^,^ e	4.8 (4.7)	1.7 (2.6)	0.0 (0.0)^b^	0.0 (0.0)^b^	**0.030**
Vegetable stock cubes	1.5 (1.4)	1.3 (1.8)^e^	3.0 (0.9)	1.3 (1.4)	1.7 (1.2)	0.3 (0.5)^b^	**0.017**
Beef gravy granules	0.7 (0.8)	0.3 (0.5)^b^	1.8 (0.4)^a^^,^ c ^,^ d ^,^ e	0.7 (1.0)^b^	0.5 (0.5)^b^	0.0 (0.0)^b^	**< 0.001**
Barbecue sauce	0.3 (0.5)	0.3 (0.5)^d^	0.0 (0.0)^d^	0.0 (0.0)^d^	1.0 (0.0)^a^^,^ b ^,^ c ^,^ e	0.0 (0.0)^d^	**< 0.001**
Soy sauce	1.0 (1.6)	0.7 (0.5)^d^	0.8 (0.4)	0.3 (0.5)^d^^,^ e	3.0 (2.7)^a^^,^ b ^,^ c ^,^ e	0.0 (0.0)^d^	**0.003**
Brown sauce	0.0 (0.0)	0.0 (0.0)	0.0 (0.0)	0.0 (0.0)	0.0 (0.0)	0.0 (0.0)	—
Frozen burgers	0.4 (1.0)	0.3 (0.5)	1.7 (1.9)^c^^,^ d ^,^ e	0.0 (0.0)^a^^,^ b	0.2 (0.4)	0.0 (0.0)^b^^,^ c ^,^ d	**0.015**
Frozen sausages	0.3 (1.3)	1.2 (2.9)	0.3 (0.5)	0.0 (0.0)	0.0 (0.0)	0.0 (0.0)	0.474
Frozen chicken sauce meal	0.0 (0.2)	0.0 (0.0)	0.0 (0.0)	0.0 (0.0)	0.2 (0.4)	0.0 (0.0)	0.426
Fish fingers	0.5 (0.9)	0.2 (0.4)	1.3 (0.8)	0.3 (0.8)	0.8 (1.3)	0.0 (0.0)	0.052
Shepherd's pie	0.0 (0.0)	0.0 (0.0)	0.0 (0.0)	0.0 (0.0)	0.0 (0.0)	0.0 (0.0)	—

*Note:* Data are presented as the mean (SD) number of gluten‐free versions available per store. Values in bold indicate statistical significance (*p* < 0.05).

^a^ Significantly more than quality supermarkets (*p* < 0.05, following *t*‐test and Bonferroni post hoc correction).

^b^ Significantly more than regular supermarkets (*p* < 0.05, following *t*‐test and Bonferroni post hoc correction).

^c^ Significantly more than budget supermarkets (*p* < 0.05, following *t*‐test and Bonferroni post hoc correction).

^d^ Significantly more than health food shops (*p* < 0.05, following *t*‐test and Bonferroni post hoc correction).

^e^ Significantly more than local shops (*p* < 0.05, following *t*‐test and Bonferroni post hoc correction).

An ANOVA identified a significant difference in availability across store categories for 14 of the gluten‐free foods, with the exceptions being fruit pies (*p* = 0.145), sausages (*p* = 0.474), chicken sauce meals (*p* = 0.426), fish fingers (*p* = 0.052), brown sauce and Shepard's pie (*p* value incalculable due to low numbers) (Table 1). Overall, regular supermarkets and health food shops stocked the greatest variety of gluten‐free versions of both wheat‐based and everyday foods. In contrast, local shops stocked only one gluten‐free version of both wheat‐based and everyday food items, of which both items represent the lowest average across the five store types. None of the five store categories stocked brown sauce or Shepard's pie in gluten‐free versions (Table 1). Following a *t*‐test and Bonferroni post hoc correction significant differences were identified in the number of gluten‐free versions available between certain store categories. Regular supermarkets had statistically significantly greater availability than at least one of the other store categories for eight of the 20 food items, including gluten‐free versions of bread, bread rolls, sweet biscuits, pizza bases, whole cake, vegetable stock, gravy, and frozen burgers. Health food shops had statistically significantly greater availability than at least one of the other store categories for six of the 20 food items, including gluten‐free versions of breakfast cereals, pasta, flour, cream crackers, barbecue sauce, and soy sauce (Table 1).

### 3.2 Availability of Gluten‐Free Foods—Comparison Between 2010 and 2021

When comparing gluten‐free product availability across all stores, of the 20 foods surveyed, overall availability of gluten‐free versions decreased from 8.2 (SD 6.8) of the 20 foods (41% of foods) per store in 2010 to 7.6 (SD 5.7, 38%) in 2021, although this was not statistically significant (*p* = 0.403). However, there were changes in availability over time specifically in regular supermarkets, where 18.0 (SD 2.0) of the 20 foods (90% of foods) were available in gluten‐free versions in 2010, but this had decreased to 14.0 (SD 1.4, 70%) in 2021 (*p* = 0.008). Although there were reductions in quality supermarkets (from mean 9.7, SD 6.5 in 2010 to 7.8, SD 5.1 in 2021, *p* = 0.363) and in local shops (from 1.8, SD 0.8 in 2010 to 0.8, SD 0.8 in 2021, *p* = 0.076), these were not statistically significant. The increases seen in availability in budget supermarkets (from mean 1.8, SD 1.0 in 2010 to 5.2, SD 5.8 in 2021, *p* = 0.153) and health food shops (from mean 9.8, SD 1.7 in 2010 to 10.3, SD 2.3 in 2021, *p* = 0.296) were also not statistically significant.

The change in thenumber of individual gluten‐free versions of wheat‐based foods between 2010 and 2021 ranged from small increases in availability of fruit pies from 0.2 to 0.4 per store to large increases of sweet biscuits from 4.8 to 11.4, respectively (Table 2).

**Table 2:** Availability (number of different versions) of gluten‐free foods in 2010 and 2021 according to store category. Data are for 30 stores overall and 6 stores for each individual store category.

Availability, mean (SD)	Stores overall			Quality supermarkets			Regular supermarkets			Budget supermarkets			Health food shops			Local shops		
	2010	2021	*p*	2010	2021	*p*	2010	2021	*p*	2010	2021	*p*	2010	2021	*p*	2010	2021	*p*
Bread	2.5 (2.7)	5.0 (7.2)	**0.024**	2.8 (2.9)	4.3 (6.6)	0.490	5.0 (1.8)	13.8 (7.4)	**0.019**	0.0 (0.0)	2.8 (4.7)	0.197	4.7 (1.4)	4.2 (7.2)	0.859	0.0 (0.0)	0.0 (0.0)	—
Bread rolls	0.9 (1.6)	1.8 (2.7)	**0.029**	2.2 (2.7)	2.0 (2.5)	0.809	2.2 (0.4)	5.2 (2.4)	**0.041**	0.0 (0.0)	1.5 (3.2)	0.304	0.0 (0.0)	0.3 (0.5)	0.175	0.0 (0.0)	0.0 (0.0)	—
Breakfast cereal	2.3 (2.7)	2.8 (3.3)	0.277	2.5 (3.6)	1.2 (1.3)	0.249	5.3 (1.0)	5.3 (2.9)	1.000	0.0 (0.0)	1.5 (2.3)	0.178	3.7 (1.9)	6.0 (3.6)	0.078	0.0 (0.0)	0.0 (0.0)	—
Pasta	2.3 (3.3)	5.8 (8.9)	**0.013**	1.5 (2.1)	2.7 (2.7)	**0.034**	3.8 (2.2)	6.3 (2.3)	0.181	0.0 (0.0)	1.3 (2.1)	0.175	6.0 (4.5)	18.5 (13.3)	0.061	0.0 (0.0)	0.0 (0.0)	—
Plain flour	0.7 (0.7)	3.6 (5.3)	**0.003**	0.8 (0.4)	1.8 (2.3)	0.332	1.0 (0.0)	2.7 (1.9)	0.080	0.0 (0.0)	1.0 (2.0)	0.275	1.5 (0.8)	11.8 (6.7)	**0.011**	0.0 (0.0)	0.7 (0.8)	0.102
Cream crackers	0.3 (0.5)	4.8 (5.2)	**< 0.001**	0.5 (0.8)	4.2 (4.5)	0.069	0.7 (0.5)	6.7 (0.8)	**< 0.001**	0.0 (0.0)	3.5 (4.5)	0.113	0.5 (0.5)	9.5 (7.3)	**0.029**	0.0 (0.0)	0.0 (0.0)	—
Sweet biscuits	4.8 (5.8)	11.4 (12.5)	**0.003**	4.8 (5.9)	10.0 (8.6)	0.151	9.2 (5.2)	21.8 (3.9)	**0.001**	0.0 (0.0)	11.8 (18.2)	0.173	10.2 (4.9)	13.3 (14.0)	0.585	0.0 (0.0)	0.0 (0.0)	—
Fruit pies	0.2 (0.5)	0.4 (1.8)	0.573	0.5 (0.8)	0.0 (0.0)	0.203	0.7 (0.5)	2.2 (3.9)	0.425	0.0 (0.0)	0.0 (0.0)	—	0.0 (0.0)	0.0 (0.0)	—	0.0 (0.0)	0.0 (0.0)	—
Pizza bases	0.3 (0.5)	0.5 (0.6)	0.083	0.3 (0.5)	0.3 (0.5)	—	0.7 (0.5)	1.0 (0.0)	0.175	0.0 (0.0)	0.5 (0.5)	0.076	0.7 (0.8)	0.8 (0.8)	0.741	0.0 (0.0)	0.0 (0.0)	—
Whole cake	2.0 (3.3)	1.6 (3.1)	0.537	2.5 (3.2)	1.5 (2.8)	0.627	4.5 (5.4)	4.8 (4.7)	0.822	0.0 (0.0)	1.7 (2.6)	0.175	2.8 (2.3)	0.0 (0.0)	**0.030**	0.0 (0.0)	0.0 (0.0)	—
Vegetable stock cubes	2.1 (1.9)	1.5 (1.4)	0.051	2.2 (1.9)	1.3 (1.8)	**0.042**	4.8 (1.2)	3.0 (0.9)	0.069	0.3 (0.5)	1.3 (1.4)	**0.041**	2.3 (1.0)	1.7 (1.2)	0.394	0.8 (0.4)	0.3 (0.5)	0.076
Beef gravy granules	0.6 (0.9)	0.7 (0.8)	0.662	0.7 (1.0)	0.3 (0.5)	0.175	1.7 (0.8)	1.8 (0.4)	0.695	0.3 (0.5)	0.7 (1.0)	0.576	0.2 (0.4)	0.5 (0.5)	0.175	0.2 (0.4)	0.0 (0.0)	0.363
Barbecue sauce	1.1 (1.2)	0.3 (0.4)	**0.001**	1.0 (0.9)	0.3 (0.5)	0.175	2.3 (0.5)	0.0 (0.0)	**< 0.001**	0.3 (0.5)	0.0 (0.0)	0.175	1.2 (1.9)	1.0 (0.0)	0.842	0.8 (0.4)	0.0 (0.0)	**0.004**
Soy sauce	0.9 (1.3)	1.0 (1.6)	0.730	0.7 (0.8)	0.7 (0.5)	1.000	1.2 (1.2)	0.8 (0.4)	0.530	0.0 (0.0)	0.3 (0.5)	0.175	2.7 (1.6)	3.0 (2.7)	0.679	0.0 (0.0)	0.0 (0.0)	—
Brown sauce	0.5 (0.8)	0.0 (0.0)	**0.002**	1.2 (1.2)	0.0 (0.0)	0.058	1.2 (0.8)	0.0 (0.0)	**0.013**	0.0 (0.0)	0.0 (0.0)	—	0.2 (0.4)	0.0 (0.0)	0.363	0.0 (0.0)	0.0 (0.0)	—
Frozen burgers	0.5 (0.7)	0.4 (1.0)	0.823	0.5 (0.8)	0.3 (0.5)	0.611	1.2 (0.8)	1.7 (1.9)	0.415	0.7 (0.5)	0.0 (0.0)	**0.025**	0.0 (0.0)	0.2 (0.4)	0.363	0.0 (0.0)	0.0 (0.0)	—
Frozen sausages	0.8 (1.3)	0.3 (1.3)	0.084	1.7 (1.4)	1.2 (2.9)	0.702	2.5 (1.2)	0.3 (0.5)	**0.015**	0.0 (0.0)	0.0 (0.0)	—	0.0 (0.0)	0.0 (0.0)	—	0.0 (0.0)	0.0 (0.0)	—
Frozen chicken meal	0.3 (0.7)	0.0 (0.2)	**0.043**	0.0 (0.0)	0.0 (0.0)	—	1.5 (0.5)	0.0 (0.0)	**0.001**	0.0 (0.0)	0.0 (0.0)	—	0.0 (0.0)	0.2 (0.4)	0.363	0.0 (0.0)	0.0 (0.0)	—
Fish fingers	0.3 (0.5)	0.5 (0.9)	0.118	0.0 (0.0)	0.2 (0.4)	0.363	1.2 (0.4)	1.3 (0.8)	0.741	0.2 (0.4)	0.3 (0.8)	0.695	0.0 (0.0)	0.8 (1.3)	0.185	0.0 (0.0)	0.0 (0.0)	—
Shepherd's pie	0.4 (0.6)	0.0 (0.0)	**0.003**	0.8 (0.8)	0.0 (0.0)	**0.042**	0.8 (0.8)	0.0 (0.0)	**0.042**	0.0 (0.0)	0.0 (0.0)	—	0.2 (0.4)	0.0 (0.0)	0.363	0.0 (0.0)	0.0 (0.0)	—

*Note:* Data are presented as the mean (SD) number of gluten‐free versions available per store. The P values represent the results of a paired t‐test comparing identical (or substitute) stores in 2010 and 2021. Values in bold indicate statistical significance (*p* < 0.05).

Paired *t*‐test comparing the number of individual gluten‐free versions between 2010 and 2021 across all store categories showed that six wheat‐based products increased in availability (bread, bread rolls, pasta, flour, crème crackers, and sweet biscuits), whereas four gluten‐free versions of everyday foods reduced in availability (barbecue sauce, brown sauce, frozen chicken sauce meal, and shepherd's pie) (Table 2). The most significant changes in availability occurred in the regular supermarket category.

### 3.3 Cost of Gluten‐Free Foods in 2021—Comparison With Standard Versions and Across Store Categories

In 2021, the cost of gluten‐free foods (average of cheapest and branded) compared to standard counterparts highlighted a significantly higher cost of 15 of the 20 gluten‐free foods. The percentage cost difference for wheat‐based gluten‐free foods over their standard equivalents ranged from 19.3% (breakfast cereals) to 181.5% (plain flour), while everyday gluten‐free foods showed a narrower range, from 1.2% (fish fingers) to 127.4% (barbecue sauce). Fruit pies and gravy were not significantly different (*p* > 0.05), while brown sauce, frozen chicken meals, and shepherd's pie were excluded from the analysis due to limited availability preventing valid comparison (Table 3).

**Table 3:** Cost (average of cheapest and branded) of gluten‐free foods compared with standard foods in 2021.

Item	*N*	Cost (pence per 100 g), mean (SD)*		*p*	% Difference
		Gluten‐free 2021	Standard 2021		
Bread	15	68.0 (56.3)	29.4 (33.9)	**0.001**	+131.3
Bread rolls	12	128.3 (122.6)	64.3 (128.8)	**< 0.001**	+99.5
Breakfast cereal	17	59.4 (24.5)	49.8 (39.4)	**0.001**	+19.3
Pasta	18	57.8 (41.5)	37.7 (25.1)	**< 0.001**	+53.3
Plain flour	19	36.6 (34.0)	13.0 (5.3)	**< 0.001**	+181.5
Cream crackers	18	154.1 (84.8)	62.1 (59.9)	**0.012**	+148.1
Sweet biscuits	17	147.0 (71.1)	56.7 (64.8)	**< 0.001**	+159.3
Fruit pies	4	77.0 (0.0)	32.1 (8.5)	0.432	+139.9
Pizza bases	9	137.2 (82.0)	63.5 (13.1)	**0.006**	+116.1
Whole cake	9	86.6 (15.5)	59.7 (16.2)	**0.045**	+45.1
Vegetable stock cubes	12	172.0 (22.8)	134.2 (48.6)	**0.011**	+28.2
Beef gravy granules	11	112.0 (10.9)	74.3 (50.0)	0.858	+50.7
Barbecue sauce	2	137.8 (30.8)	60.6 (25.0)	**< 0.001**	+127.4
Soy sauce	15	144.0 (49.4)	95.8 (43.2)	**0.007**	+50.3
Brown sauce	0	—	—	—	—
Frozen burgers	6	87.2 (30.4)	76.9 (23.9)	0.859	+13.4
Frozen sausages	3	54.4 (17.9)	44.7 (23.4)	0.450	+21.7
Frozen chicken sauce meal	0	—	—	—	—
Fish fingers	8	95.2 (21.7)	94.1 (96.9)	**0.001**	+1.2
Shepherd's pie	0	—	—	—	—

*Note:* Data are presented as the cost per 100 g, except for white bread rolls and whole cake, which are presented as the cost per unit (i.e., per roll, per cake). The data compare the cost of gluten‐free and standard versions by comparing the average of branded and cheapest versions in each store, where available. Therefore, as a result of the lower availability of some versions, data were not available for all stores. Currency conversion rates at the time of data collection were £1 (100 pence) = US $1.42 = €1.16.

The cost (average of branded and cheapest) of nine gluten‐free foods varied significantly across store categories. Both regular and budget supermarkets generally offered gluten‐free products at the lowest prices of the five store categories (Table 4). For example, the cost of bread rolls, breakfast cereal, pasta, flour, cream crackers, pizza bases, whole cake, soy sauce and fish fingers were significantly more expensive at quality supermarkets, health food stores or local shops compared to the lowest‐priced stores, specifically regular or budget stores (Table 4).

**Table 4:** Cost (average of branded and cheapest) of gluten‐free foods according to store category in 2021.

Cost (pence per 100 g), mean (SD)	Overall cost (all stores)	(a) Quality supermarket	(b) Regular supermarket	(c) Budget supermarket	(d) Health food shops	(e) Local shops	*p*
Bread	68.0 (56.3)	109.7 (100.2)	38.5 (2.9)	58.7 (3.8)	77.6 (27.0)	—	0.278
Bread rolls	132.2 (118.3)	153.1 (28.7)	68.0 (6.8)^d^	87.5 (17.7)	338.3 (225.2)^b^	—	**0.012**
Breakfast cereal	69.9 (50.7)	136.4 (98.9)	51.1 (16.8)	33.7 (1.2)	73.7 (25.9)	—	**0.037**
Pasta	59.2 (40.8)	72.3 (42.0)	30.9 (12.5)^d^	12.0 (0.0)^d^	92.4 (33.0)^b^^,^ c	—	**0.006**
Flour	35.5 (32.5)	22.2 (5.6)	13.6 (5.1)	17.0 (0.0)	59.8 (42.5)	60.9 (32.3)	**0.033**
Cream crackers	153.7 (82.5)	179.9 (36.5)	102.1 (18.3)^d^	85.7 (11.8)	221.8 (105.9)^b^	—	**0.015**
Sweet biscuits	162.0 (107.0)	242.0 (173.7)	104.5 (23.9)	94.2 (13.8)	191.5 (55.1)	—	0.093
Fruit pies	—	—	—	—	—	—	—
Pizza bases	200.1 (187.6)	206.0 (0.0)	99.1 (1.4)^d^	96.8 (5.6)^d^	426.1 (255.0)^b^^,^ c	—	**0.014**
Whole cake	86.6 (15.5)	106.8 (14.2)^c^	85.4 (8.5)	69.4 (2.7)^a^	—	—	**0.018**
Vegetable stock cubes	191.3 (47.8)	242.7 (90.3)	160.7 (15.8)	173.3 (32.2)	196.2 (31.5)	230.0 (26.5)	0.074
Beef gravy granules	129.6 (44.1)	120.6 (0.0)	112.6 (13.3)	104.9 (0.0)	186.0 (69.9)	—	0.060
Barbecue sauce	128.4 (26.9)	137.8 (30.8)	—	—	125.3 (27.8)	—	0.609
Soy sauce	143.5 (46.2)	147.3 (46.7)	109.9 (13.1)^d^	104.0 (0.0)	182.1 (42.9)^b^	—	**0.020**
Brown sauce	—	—	—	—	—	—	—
Frozen burgers	87.2 (30.4)	104.7 (48.4)	67.8 (1.9)	—	110.4^f^	—	0.362
Frozen sausages	54.4 (17.9)	75.0^f^	44.1 (0.0)	—	—	—	—
Frozen chicken sauce meal	—	—	—	—	210.3 (0.0)	—	—
Fish fingers	101.2 (27.2)	101.4^f^	82.8 (0.7)	101.2^f^	147.2 (3.5)	—	**< 0.001**
Shepherd's pie	—	—	—	—	—	—	—

*Note:* Data are presented as the cost per 100 g, except for white bread rolls and whole cake, which are presented as the cost per unit (i.e., per roll, per cake). The currency conversion rates at the time of data collection were £1 (100 pence) = US $1.42 = €1.16. Values in bold indicate statistical significance (*p* < 0.05).

^a^ Significantly more expensive than quality supermarkets (*p* < 0.05, following *t*‐test and Bonferroni *post hoc* correction).

^b^ Significantly more expensive than regular supermarkets (*p* < 0.05, following t‐test and Bonferroni *post hoc* correction).

^c^ Significantly more expensive than budget supermarkets (*p* < 0.05, following *t*‐test and Bonferroni *post hoc* correction).

^d^ Significantly more expensive than health food shops (*p* < 0.05, following *t*‐test and Bonferroni *post hoc* correction).

^e^ Significantly more expensive than local shops (*p* < 0.05, following *t*‐test and Bonferroni *post hoc* correction).

^f^ Indicates availability of foods were limited such that SD could not be calculated.

### 3.4 Cost of Gluten‐Free Foods—Comparison Between 2010 and 2021

The cost (average of branded and cheapest) of several gluten‐free versions of foods differed significantly between 2010 and 2021, with seven foods increasing in cost and only one decreasing. Between 2010 and 2021, notable increases in cost were observed for bread rolls rising by 95%, pizza bases by 75.7%, fruit pies by 71.1%, fish fingers by 33.5%, beef gravy granules by 28.8% and crème crackers by 19.4%, whereas whole cake decreased by 54.1% (Table 5).

**Table 5:** Cost of gluten‐free foods (the average of the cheapest and branded version) across all stores comparing 2010 and 2021, together with inflation‐adjusted anticipated costs for 2021.

	*N*	Cost (pence per 100 g), mean (SD)		*p*	Anticipated 2021 cost with CPI added to 2010 cost (pence per 100 g)^a^
		All stores 2010	All stores 2021		
Bread	12	71.1 (42.4)	70.0 (63.3)	0.891	74.7
Bread rolls	8	47.9 (9.1)	93.4 (47.4)	**0.013**	50.3
Breakfast cereal	15	59.5 (24.5)	77.2 (52.8)	0.095	62.5
Pasta	15	55.0 (13.6)	61.3 (36.5)	0.397	57.8
Plain flour	15	22.2 (6.3)	33.6 (34.0)	0.192	23.3
Cream crackers	9	111.8 (24.2)	133.5 (22.3)	**0.047**	117.4
Sweet biscuits	11	140.3 (57.6)	181.7 (118.1)	0.072	147.3
Fruit pies	2	45.0 (0.0)	77.0 (0.1)	**0.001**	47.3
Pizza bases	8	105.2 (33.8)	184.8 (103.5)	**0.042**	110.5
Whole cake	6	197.5 (49.7)	90.6 (14.9)	**0.001**	207.4
Vegetable stock cubes	18	135.1 (51.3)	196.3 (47.7)	**< 0.001**	141.9
Beef gravy granules	9	88.2 (31.5)	113.6 (11.5)	**0.045**	92.6
Barbecue sauce	5	83.6 (41.4)	124.1 (34.3)	0.133	87.8
Soy sauce	11	144.0 (25.8)	158.2 (48.6)	0.259	151.2
Brown sauce	0	—	—	—	—
Frozen burgers	4	55.4 (7.9)	85.6 (35.6)	0.150	58.2
Frozen sausages	3	56.0 (13.8)	54.4 (17.9)	0.752	58.8
Frozen chicken sauce meal	0	—	—	—	—
Fish fingers	5	62.0 (4.6)	82.8 (0.7)	**< 0.001**	65.1
Shepherd's pie	0	—	—	—	—

*Note:* Data are presented as the cost per 100 g, except for white bread rolls and whole cake, which are presented as the cost per unit (i.e., per roll, per cake). *p* values are the result of a paired *t*‐test between identical (or substitute stores) in 2010 and 2021. Values in bold indicate statistical significance (*p* < 0.05).

^a^ The inflation rate of 1.05% calculated over an 11‐year period was applied to the 2010 cost to arrive at the anticipated 2021 inflation‐adjusted cost.

Notable differences were observed when comparing the 2021 cost to the inflation‐adjusted anticipated costs for 2021, with 14 foods being more expensive than anticipated by inflation. For example, the cost of bread rolls exceeded anticipated inflated cost by 85.7%, fruit pies by 62.8%, pizza bases by 67.2%, vegetable stock by 38.3% and gravy by 22.7%. In contrast, whole cake was 56.3% lower than the anticipated inflation adjusted cost (Table 5).

### 3.5 Cost Ratio of Gluten‐Free and Standard Foods—Comparison Between 2010 and 2021

The cost ratio between a gluten‐free food item and standard counterpart in the same store was calculated for 2010 and for 2021. Cost ratios were then compared using a paired t‐test between identical stores that sold a gluten‐free and standard version of a food item in both 2010 and 2021.

For a small number of food items, the cost ratio between gluten‐free foods and standard counterparts reduced between 2010 and 2021. For example, the cost ratio for bread decreased significantly from 4.5‐fold higher (SD ± 1.3) in 2010 to 3.0 (1.2) in 2021, for pasta from 2.5‐fold higher (0.5) to 1.6 (0.6), for sweet biscuits 7.1‐fold higher (2.6) to 4.3 (1.4) and for fruit pies 3.2‐fold higher (0.0) to 2.0 (0.0) (Table 6). No foods increased in cost ratio between 2010 and 2021.

**Table 6:** Cost ratio of gluten‐free and standard versions comparing 2010 and 2021.

		Ratio of cost of gluten‐free foods to standard (gluten‐containing) foods		
	*N*	2010	2021	*p* value
Bread	12	4.5 (1.3)	3.0 (1.2)	**0.001**
Bread rolls	8	4.4 (1.2)	3.9 (1.5)	0.482
Breakfast cereal	14	2.2 (0.5)	1.6 (0.8)	0.053
Pasta	12	2.5 (0.5)	1.6 (0.6)	**0.001**
Plain flour	13	3.1 (1.0)	2.3 (1.2)	0.169
Cream crackers	7	4.3 (1.0)	4.0 (1.4)	0.451
Sweet biscuits	8	7.1 (2.6)	4.3 (1.4)	**0.001**
Fruit pies	2	3.2 (0.0)	2.0 (0.0)	**0.002**
Pizza bases	5	2.3 (0.4)	2.1 (0.7)	0.362
Whole cake	6	1.7 (0.6)	1.5 (0.2)	0.434
Vegetable stock cubes	11	1.1 (0.4)	1.3 (0.2)	0.418
Beef gravy granules	9	1.9 (0.9)	1.8 (0.7)	0.592
Barbecue sauce	2	1.0 (0.0)	2.4 (0.5)	0.152
Soy sauce	9	1.5 (0.3)	1.5 (0.5)	0.904
Brown sauce	0	—	—	—
Frozen burgers	4	1.2 (0.2)	1.4 (0.6)	0.700
Frozen sausages	3	2.5 (0.3)	1.4 (0.7)	0.209
Frozen chicken sauce meal	0	—	—	—
Fish fingers	5	1.4 (0.1)	1.4 (0.4)	0.985
Shepherd's pie	0	—	—	—

*Note:* Data are presented as the ratio of the cost of gluten‐free and standard versions by comparing the average of branded and cheapest versions in the same store at both timepoints, where available. Due to the lower availability of some gluten‐free versions, data were not available for all foods at all stores at both timepoints, and therefore the number of stores providing data are presented. *p* values are the result of a paired *t*‐test between identical stores that carried a gluten‐free and standard version of a food item in both 2010 and 2021. Values in bold indicate statistical significance (*p* < 0.05).

## 4 Discussion

Adherence to a lifelong gluten‐free diet remains the only treatment for people with coeliac disease, and yet limited availability and high cost of gluten‐free foods remains a challenge. The present study demonstrated that overall, gluten‐free food availability remains limited and indeed has declined over a decade, whilst costs of gluten‐free foods continued to increase and remain higher than standard, gluten‐containing versions. These disparities in availability and cost may disproportionately impact individuals from lower socioeconomic backgrounds, as well as elderly, disabled, chronically ill or less mobile populations who rely on purchasing gluten‐free products [21]. More positive findings include some evidence of increased availability in budget supermarkets from 2010 to 2021 and a reduction in the relative cost ratio compared to standard versions over time. This study is the first in the UK to directly replicate an earlier investigation using an identical methodological approach to evaluate the availability and cost of gluten‐free foods more than a decade later.

### 4.1 Availability of Gluten‐Free Foods

The findings have indicated a decrease in availability of gluten‐free foods over the past 11 years overall, although not statistically significant, and specifically at regular supermarkets. Notably, however, the availability of six gluten‐free versions of wheat‐based foods increased across all store categories, while the availability of four everyday gluten free products declined. Between store categories, regular and quality supermarkets and health food shops maintained the greatest availability of the five store categories respectively, coinciding with previous literature [17, 18]. Although online stores were not examined in the present study, it is acknowledged that previous literature have reported notably higher availability (up to 100%) of gluten‐free products for purchase online when compared to supermarkets, convenience stores and health food shops [17, 18, 20].

The purchase of commercially available gluten‐free foods is largely attributable to the rising awareness and diagnosis of coeliac disease, alongside the withdrawal of gluten free prescriptions in several UK National Health Service Trusts [21]. This is reflected in the expansion of the UK gluten‐free market, which is now valued at £835 million annually [30]. These developments have likely influenced consumer purchasing behaviours and product availability through the dynamics of supply and demand, influencing greater availability of wheat‐based gluten‐free foods and a reduction in everyday gluten‐free foods across all store classifications since 2010 [31, 32, 33]. This is especially relevant as wheat‐based foods such as pasta, flour, and bread, all previously available on prescription in England, have all increased in overall availability and remain staples in UK households, while breads and flours remain limited on prescription on Scotland, Wales and Northern Ireland [13, 19]. In particular, bread is purchased by 99.8 percent of all general households and is perceived as essential by patients with coeliac disease to maintain a ‘normal diet' [17, 19, 34]. Accordingly, regular supermarkets reported an increase in availability of wheat‐based items, including bread and bread rolls, despite an overall decline in total gluten‐free product availability. These changes may reflect evolving consumer preferences, with an observed increase in biscuit availability potentially corresponding with greater consumer interest, and a comparatively reduced presence of gluten‐free cakes in health food stores relative to 2010 [19, 35, 36].

Conversely, the overall availability and range of everyday gluten‐free products has decreased across all stores since 2010, including barbecue sauce, brown sauce, frozen chicken sauce meal and Shepard's pie, which are no longer stocked at regular supermarkets. This decline may reflect decreasing consumer interest, driven by perceived high cost or variable organoleptic properties [9, 19]. Additionally, increasing consumer awareness of the potential health risks associated with ultra‐processed foods (UPFs), combined with the substantial proportion of commercially available gluten‐free products classified as UPFs, may influence purchasing behaviours of these examples of food products.

There was some evidence of improvements in the availability and range of gluten‐free foods were observed in budget supermarkets (17% increase, although not statistically significant). The improvement in availability may be attributed to advancements in manufacturing, processing, and production techniques, which have likely reduced production costs and improved affordability, thereby facilitating broader distribution to budget supermarkets [9, 37, 38, 39]. A recent report confirmed the expansion of supermarket own‐label gluten‐free products, which may contribute towards the increased availability at budget supermarkets [40]. The increased range and availability of wheat‐based gluten‐free products in budget supermarkets represents a positive development for lower socioeconomic populations with coeliac disease needing to adhere to a strict gluten‐free diet. Further investigation is warranted to examine the availability of gluten‐free foods in budget supermarkets across various geographical locations with differing levels of deprivation, as previous literature has highlighted inconsistencies in availability that may be dependent on store location [17, 18, 20].

The very limited availability in local shops may have decreased further due to cost constraints, reduced consumer demand, and limited shelf space. This may disproportionately affect lower socioeconomic groups some of whom may rely on local shops, may experience greater food insecurity or have limited access to adequate nutrition, increasing the risk of poor adherence to gluten‐free diet and adverse health outcomes [17, 21, 41].

### 4.2 Cost of Gluten‐Free Foods

Overall, the cost of gluten‐free foods remains substantially higher than that of standard gluten‐containing counterparts, with prices increasing since the initial study conducted in 2010 and exceeding inflation‐adjusted projections. However, some people with coeliac disease in the UK continue to access gluten‐free foods via prescription and may therefore be less directly affected by increased retail costs [21, 42]. In England, prescribing is now highly restricted to bread and flour mixes only, with many local commissioning areas discontinuing prescriptions entirely [13, 21, 42, 43]. Consequently, access to gluten‐free foods on prescription is variable and increasingly limited, requiring a growing number of individuals to purchase these products themselves [13, 21, 42]. In addition, those without coeliac disease but who follow a gluten‐free diet and who are not eligible for gluten‐free prescriptions, such as people with gluten sensitivity or those following a low FODMAP diet, may also experience problems if the elevated cost. Notably, however, the relative cost difference between gluten‐free and standard foods decreased over the same period.

Unsurprisingly, regular and budget supermarkets generally offered lower cost gluten‐free foods compared with quality supermarkets, health food shops, and local shops, consistent with our findings from 2010. Previous literature has consistently highlighted that gluten‐free foods are more expensive to purchase online compared to physical retail stores [17, 18, 20].

Since 2010, prices increased for four gluten‐free wheat‐based products, with availability increasing for two, potentially indicating higher consumer demand and expanded supply [44]. Five products exhibited price increases despite stable or limited availability, suggesting influences such as rising production costs or supply chain constraints [31, 44]. Conversely, whole cake declined in both cost and availability, possibly reflecting reduced demand.

Despite rising gluten‐free product costs exceeding inflation‐adjusted estimates, the percentage cost difference from standard items for ‘everyday gluten‐free products' remained relatively stable (2%–124% in 2010 vs. 1%–127% in 2021). In contrast, gluten‐free wheat‐based product costs declined substantially (76%–518% vs. 19%–182%), aligning with recent reports of 118% [36] and 159% [37] higher average costs for such items.

An overall reduction in the price ratio between gluten‐free foods and standard counterparts was observed between 2010 and 2021. Similarly, a longitudinal study conducted in the U.S. over a decade reported a decline in the cost of gluten‐free products compared to their standard counterparts, adjusted for inflation [20]. In part this may be driven by greater increases in standard food prices [45, 46]. This represents progress in reducing the cost disparity between gluten‐free and standard foods. The decrease in price ratio contrasts with a 4‐year longitudinal study, which highlighted an increase in the cost ratio of 15 out of 21 products between 2015 and 2019, with only six showing a decrease [19]. These contrasting findings may reflect differences in study design, including the time points and periods of sampling, variations in the products sampled, the types of stores analysed, and the geographical locations within the UK.

Importantly, the reducing cost ratio differential identified in the present study may reflect a broader market shift toward improved affordability. Such developments may be partially driven by innovations and advancements in ingredient sourcing, manufacturing, processing, and production methods that have enabled more cost‐efficient production [38, 39]. The specific decreases in the price ratio differential between 2010 and 2021 observed for bread and pasta, both of which are staple foods in the UK, may be attributed to several factors including greater consumer demand, improved manufacturing efficiency, expanded retail availability, and increased market competition [34, 35, 47, 48]. These products may benefit from more streamlined supply chains, economies of scale, and greater prioritisation by retailers and manufacturers due to their broader appeal [49, 50].

Future research is essential to evaluate the impact of rising inflation and the cost‐of‐living crisis on the cost ratio, particularly in light of increases in food and drink prices and CPI in the UK since September 2021, when these data were collected [51, 52]. Those without coeliac disease, such as individuals with gluten sensitivity or following a low FODMAP diet, also face ongoing cost and availability barriers.

### 4.3 Strengths and Limitations

A key strength of this study is the replication of the 2010 methodology, enabling direct comparisons often lacking in cross‐sectional designs, although minor methodological differences may have influenced the results. Another strength of this study is the use of food‐specific CPI to adjust for inflation, allowing more accurate estimation of expected price changes over 11 years and comparison with actual pricing trends.

A key limitation of this study is that store observations were conducted exclusively in Greater London, which may not reflect availability and cost in other regions of the UK or internationally. London's higher cost of living may also introduce a pricing bias, potentially affecting food costs, however, direct comparisons with standard versions in the same store and with 2010, both of which were also in London should minimise the impact of this on data interpretation [53]. Food access, store types, store sizes, and product availability vary across the UK [17]. However, the sampling included five store categories and a wide range of boroughs with varying local populations. This study did not assess the availability or cost of gluten‐free foods online, although previous studies have analysed online availability of gluten‐free foods [17, 18, 20]. Including online retail data in future research would offer a more comprehensive evaluation, particularly for individuals with limited mobility or store access. This is increasingly relevant as recent data indicated that online grocery shopping has become a well‐established component of consumer behaviour in the UK, rising from 16% in 2010 to 36% in 2023, as many shoppers now adopt a hybrid approach combing in‐store and online purchases [27, 54, 55].

## 5 Conclusion

The availability of gluten‐free foods remains limited in the UK, and cost continue to exceed both standard gluten‐containing foods and inflation. These factors may contribute to the ongoing challenges with adherence to a strict gluten‐free diet. Encouragingly, the increased availability of some gluten‐free foods in budget supermarkets and the decrease in the cost ratio across all stores over the past decade represent positive developments, particularly for people with coeliac disease for whom permanent adherence to a gluten‐free diet is compulsory cornerstone of treatment. Nonetheless, until gluten‐free foods reach equivalence with their gluten‐containing counterparts in terms of both availability and cost, access will continue to be regarded as limited and inequitable. Future research should explore whether increased availability and affordability of gluten‐free foods lead to improved dietary adherence, especially among vulnerable groups such as older people and those from lower socioeconomic groups.

## Author Contributions

K.W. conceived the study, K.W. designed the study. L.K., U.M., J.S., undertook data collection. H.M., L.K., U.M., K.W. analysed and interpreted the data. H.M. and K.W. wrote the manuscript. All authors approved the manuscript for publication.

## Funding

The authors received no specific funding for this work.

## Ethics Statement

The authors have nothing to report.

## Conflicts of Interest

The other authors declare no conflicts of interest. K.W. has received research grants related to diet and gut health and disease from Almond Board of California, Danone, and International Nut and Dried Fruit Council and has received speaker fees from Danone and Yakult. K.W. is the holder of a joint patent to use volatile organic compounds as biomarkers in irritable bowel syndrome (PCT/GB2020/051604). K.W. receives royalties from Wiley Publishing in relation to academic textbooks on nutrition and dietetics.

## Artificial Intelligence Generated Content (AIGC)

Artificial Intelligence was not used in any way during the analysis or interpretation of data or writing of the manuscript.
